# Using natural language processing to identify the status of homelessness and housing instability among serious illness patients from clinical notes in an integrated healthcare system

**DOI:** 10.1093/jamiaopen/ooad082

**Published:** 2023-09-22

**Authors:** Fagen Xie, Susan Wang, Lori Viveros, Allegra Rich, Huong Q Nguyen, Ariadna Padilla, Lindsey Lyons, Claudia L Nau

**Affiliations:** Department of Research and Evaluation, Kaiser Permanente Southern California, Pasadena, CA 91101, United States; Department of Research and Evaluation, Kaiser Permanente Southern California, Pasadena, CA 91101, United States; Department of Research and Evaluation, Kaiser Permanente Southern California, Pasadena, CA 91101, United States; Department of Research and Evaluation, Kaiser Permanente Southern California, Pasadena, CA 91101, United States; Department of Research and Evaluation, Kaiser Permanente Southern California, Pasadena, CA 91101, United States; Department of Research and Evaluation, Kaiser Permanente Southern California, Pasadena, CA 91101, United States; Department of Research and Evaluation, Kaiser Permanente Southern California, Pasadena, CA 91101, United States; Department of Research and Evaluation, Kaiser Permanente Southern California, Pasadena, CA 91101, United States

**Keywords:** homeless, residential instability, natural language processing, electronic health record, serious illness

## Abstract

**Background:**

Efficiently identifying the social risks of patients with serious illnesses (SIs) is the critical first step in providing patient-centered and value-driven care for this medically vulnerable population.

**Objective:**

To apply and further hone an existing natural language process (NLP) algorithm that identifies patients who are homeless/at risk of homeless to a SI population.

**Methods:**

Patients diagnosed with SI between 2019 and 2020 were identified using an adapted list of diagnosis codes from the Center for Advance Palliative Care from the Kaiser Permanente Southern California electronic health record. Clinical notes associated with medical encounters within 6 months before and after the diagnosis date were processed by a previously developed NLP algorithm to identify patients who were homeless/at risk of homelessness. To improve the generalizability to the SI population, the algorithm was refined by multiple iterations of chart review and adjudication. The updated algorithm was then applied to the SI population.

**Results:**

Among 206 993 patients with a SI diagnosis, 1737 (0.84%) were identified as homeless/at risk of homelessness. These patients were more likely to be male (51.1%), age among 45-64 years (44.7%), and have one or more emergency visit (65.8%) within a year of their diagnosis date. Validation of the updated algorithm yielded a sensitivity of 100.0% and a positive predictive value of 93.8%.

**Conclusions:**

The improved NLP algorithm effectively identified patients with SI who were homeless/at risk of homelessness and can be used to target interventions for this vulnerable group.

## Introduction

Housing instability, the perceived risk of losing one’s apartment, room, or home, and homelessness are important social determinants of health (SDOH). ^1^^,^^2^ Housing instability and homelessness bring about many challenges to a person’s daily life ^3^ and can result in poor physical health outcomes, decreased life expectancy, and high treatment costs. ^4^^,^^5^ On the other hand, patients who are experiencing serious, often life-limiting illnesses such as cancer, heart failure, or chronic obstructive pulmonary disease need intense medical or palliative care and are often among the patients with the highest levels of healthcare utilization and associated costs. ^6^^,^^7^ Efficiently identifying the social risk and social needs that exacerbate and complicate a person’s existing illnesses is a critical first step in providing appropriate, patient-centered, and value-driven care to the seriously ill population.

Although the International Classification of Diseases 10th revision (ICD-10) coding system accommodates documentation of SDOH, ^8^ healthcare providers often document SDOH in electronic health records (EHRs) via free text format rather than structured codes. Accurately identifying SDOH typically requires utilizing the free text format narrative descriptions embedded in clinical notes. Due to the large volume of clinical notes, the traditional approach of extracting information from clinical notes—a labor-intensive and time-consuming manual chart review—is infeasible and would be prohibitively costly for identifying homeless patients or patients at risk of homelessness among a large patient group such as the serious illness (SI) population.

Natural language processing (NLP) is a field of computer-based methods aimed at standardizing and analyzing free text data elements. ^9–11^ NLP converts information residing in natural language—clinical notes generated during the provision of care—into a more structured format and has been used for medical research and patient care management purposes. ^12^^,^^13^ Several NLP applications have aimed to extract information on SDOH from various EHRs systems. ^14–18^ Recently, the study team at Johns Hopkins Health System (JHHS), Kaiser Permanente Southern California (KPSC), and Kaiser Permanente Mid-Atlantic States (KPMAS) collaborated to develop an NLP algorithm to identify residential instability from the Epic-based EHRs. ^19^ The development at the KPSC site was based on a sample of 300 clinical notes from patients with recent emergency department (ED) or hospital encounters. This small sample was likely not representative of patients living with SI, as their daily lives and health care use are likely markedly different from that of the population of ED visit users because of their SI. The purpose of this study was to test and improve the robustness and generalizability of our initial NLP algorithm in identifying homelessness or being at risk of homelessness of a highly vulnerable patient population at KPSC.

## Methods

### Study setting and population

KPSC is an integrated healthcare system with 15 hospitals and more than 250 medical offices that provides medical services to over 4.8 million members. KPSC membership broadly represents the demographic composition of its Southern California service region. ^20^ KPSC’s comprehensive EHR system captures all information recorded during the provision of care. EHR data include structured and unstructured data. Structured data are data that can be extracted from standardized data fields and include ICD diagnosis and procedure codes. Unstructured data include free-text clinical notes that healthcare providers enter in note fields during healthcare encounters. The study protocol was reviewed and approved by the KPSC Institutional Review Board with a waiver of the requirement for informed consent.

Patients diagnosed with SIs between 2019 and 2020 were identified by using an adapted list of SI ICD-10 codes developed by the Center for Advance Palliative Care (CAPC). The CAPC list was developed by a workgroup of healthcare providers and payers and combined findings with the National Committee for Quality Assurance (NCQA) Advanced Illness Value Set that was released in 2020. ^21^ The CAPC list was adapted by KPSC Palliative Care leadership to reflect KPSC’s definition of SI.

### Data preparation

The analytic sample was limited to patients with a diagnosis of SI. The early diagnosis date of SI was defined as the index date. The clinical notes associated with any encounters within 6 months prior to/after the index date were extracted from the KPSC EHR system. Patient and discharge instructional notes were excluded as these notes usually provide general warnings, instructions, or recommendations rather than specific details of the patient’s social conditions and needs. The extracted clinical notes were preprocessed including, (1) deleting special and nonword or digital characters, (2) correcting mistyped or concatenated words, (3) standardizing abbreviated words, and (4) sentence separation and tokenization. ^10^

### Homelessness and housing instability feature collection

A set of homelessness and housing instability-related keywords and their corresponding linguistic phrases, patterns, and variants were developed in our previous study ^19^ based on the standard code terminology (eg, diagnosis codes, procedure codes, etc.), literature reviews, ^14–18^ and study notes. ^19^ In this study, we further refined the list and added additional terms identified during the iterative process of prediction and chart review. In addition, a set of negation terms, nonpatient’s self-terms describing someone other than the patient (eg, a family member being homeless), and history terms describing past events rather than recent or current living situations were excluded. The terms were constructed to PhraseMatcher patterns for matching processing in spaCy. ^22^ Examples for each category were summarized in Table S1.

### Development of the NLP algorithm

The NLP algorithm that was previously developed to predict residential instability and homelessness ^19^ was based on the JHHS linguistic patterns of homeless/housing instability and the manual annotation of a randomly selected sample of 300 patients in the KPSC. We conducted chart review of a small randomly selected sample of patient charts (*N* = 30) of seriously ill patients that had been identified as patients who were homeless/at risk of homelessness by our initial NLP algorithm and found that the precision in this new population had dropped to 60%. To improve the performance of the algorithm, we conducted an additional 4 rounds of chart review where we manually reviewed patient charts and adjusted the phrases/patterns of the NLP algorithm before testing the algorithm again. We found that patients with SI had some different linguistic patterns for recording homelessness. The phrases and patterns that were added or dropped are available in Table S2. The first 3 rounds of prediction and chart review were used to train and adapt the linguistic patterns and variants iteratively based on the manual chart review findings. The last round was used to validate the performance of the refined algorithms. Each of the 3 training datasets contained 30 randomly selected patients that were classified by the existing algorithm as being homeless/experiencing housing instability and 30 patients that were identified as not being homeless/experiencing housing instability. The validation dataset contained 100 patients that were identified by the existing NLP algorithm as being homeless/experiencing housing instability cases.

### NLP training

Using the EntityRuler module of the spaCy 2.3 Python toolkit, we constructed a set of spaCy PhraseMatcher patterns to capture negation terms, nonpatient’s self-terms (terms such as “my son is living on the street”), history terms, and positive homeless and housing instability terms. These patterns included word lemmas and base forms to account for morphological variations (eg, singular and plural forms) embedded in spaCy. The process searched and refined these patterns for each sentence of the training datasets to match with the results of the reference standards derived through chart review sequentially using the following steps for each note:

1. Search the note to see whether it contained at least one of the keywords, including “lahsa” (Los Angeles Homelessness Services Authority), “hopics” (homelessness outreach program integrated care system), and “coordinated entry system,” which are major homeless programs/systems in Los Angeles County where the majority of our patients live. If any of the 3 keywords were found, the status of homeless/housing instability was defined as “Yes,” and the process moved to the next note.
2. Search the sentences within the note to determine whether they contained or mentioned any potential homeless/housing instability-related keywords or phrases (see details in Table S1). If no keyword within any sentence was detected, the status of homeless/housing instability was defined as “No” for this note and the processing moved to the next note.
3. Search sentences within the note that contained a negated description of homelessness. If such a phrase was detected, the status of homeless/housing instability was defined as “No” for this note and the processing moved to the next note. Examples of negated descriptions and descriptions not related to homelessness are shown in Table S3.
4. Search sentences within the note that were associated with a term relating to a history of homelessness/housing instability. If a phrase relating to homelessness/housing instability history was detected, the status of homeless/housing instability was related to a past event rather than the current situation, and therefore this instance was defined as “No.” The processing stopped here and moved to the next note. Examples are shown in Table S3.
5. Search sentences within the note with a positive homeless/housing instability term. If a positive term was detected, it was then examined whether the term was associated with a gender description, such as “he,” “she,” “him,” “her,” “male,” or “female.” If a gender term was described, but it did not match the patient’s gender documented in the administrative structure database (eg, “he is homeless” but the patient’s gender is female in the structured data), this positive term description was considered a negative result because it was likely to relate to another person (often a relative) rather than the patient themselves. Otherwise, the status of homeless/housing instability was defined as “Yes” and moved to the next note. Examples are shown in Table S3.

### Classifying homeless/housing instability status at the patient level

Patients’ clinical notes may contain multiple references to homelessness/housing instability. The status of homeless/housing instability at the patient level was classified as “Yes” if at least one instance in the clinical notes for each individual was deemed as “Yes.” Otherwise, it was classified as “No.”

### NLP performance evaluation

For individuals included in the validation dataset, computerized algorithm results were compared to the manual chart review. The proportions of true positive (TP), false positive (FP), true negative (TN), and false negative (FN) cases were used to estimate the sensitivity, specificity, positive predictive value (PPV), negative predictive value (NPV), and the overall F-score—a measure of overall model fit. Sensitivity was defined as the proportion of cases correctly classified by the computerized algorithm (TP) among all cases (TP+FN) ascertained by chart review. Specificity was defined as the proportion of cases correctly classified as nonhomeless/experienced housing instability by the computerized algorithm (TN) among all individuals who were not homeless/experienced housing instability (TN+FP) according to chart review. PPV was defined as the proportion of positive cases correctly classified (TP) among all those classified by the computerized algorithm as being positive cases (TP+FP). NPV was defined as the proportion of cases correctly classified as nonhomeless/housing instability (TN) among all nonhomeless/housing instability cases classified by the computerized algorithm (TN+FN). The overall accuracy F-score for each comparison was calculated via the standard formula as (2×PPV×sensitivity)/(PPV+Sensitivity). ^23^

### Chart review and reference standard

A team of researchers performed a full medical chart review of records within 6 months prior to/after the index date to determine the residential instability status for the selected study sample and documented the reasons for assigning each candidate a “Yes” or “No” labels and the supporting evidence for each assignment. For cases where a researcher was not able to determine the homelessness/housing instability status and for all cases where the NLP algorithm disagreed with the findings of the manual chart review of the study, the study team jointly reviewed and adjudicated the case. The chart review results of the training dataset were used to further improve the NLP algorithm while the chart review results of the validation dataset served to assess the final accuracy of the NLP algorithm.

### NLP algorithm implementation

The refined algorithm was implemented via spaCy ^22^ on a Linux server to process the entire set of clinical notes. The algorithm created the output of the final homeless/housing instability status among the patients with SI for further descriptive and crude logisitic regression analysis by demographic characteristics and ED utilization within 1 year before and after the index date.

## Results

The performance of the NLP algorithm against the validation dataset was summarized in Table 1. A total of 64 of 100 patients in the validation dataset were classified as homeless/experiencing housing instability. The manual chart review confirmed 60 of these 64 patients as true cases of homelessness/experiencing housing instability. The NLP algorithm achieved a sensitivity of 100.0%, specificity of 90.0%, PPV of 93.8%, NPV of 100.0%, and an F-score of 0.97.

**Table 1.** Comparison of the performance of the homeless/housing instability status assigned by the computerized algorithm against manual chart reviews on validation dataset.

Computerized algorithm classification	Chart review classification		
	Yes	No	All
Yes	60	4	64
No	0	36	36
All	60	40	100
Performance			
Sensitivity	100.0%		
Specificity	90.0%		
PPV	93.8%		
NPV	100.0%		
F-score	0.97		

Among the 206 993 KPSC patients with SI, 1737 (0.84%) were identified as being homeless/experiencing housing instability by the final NLP algorithm. The demographic characteristics of the study population by the status of homeless/housing instability were summarized in Table 2. The differences in age and ED visits between the 2 homeless/housing instability groups were statistically significant. Slightly more than half of the patients for whom we did not find evidence of homelessness/housing instability were female (52.4%), while the percentage was reversed in the homeless/housing instability group (male, 51.1%). The median age was 68.0 and 61.0 years among patients without homeless/housing instability and patients with homeless/housing instability, respectively. The age group 45-64 years had the highest percentage (44.7%) of all homeless patients. We found that 16.7% of patients with an indication of homelessness were 75 years older. Among patients with homeless/housing instability, 44.5% were Non-Hispanic White, 31.1% were Hispanic, 18.7% were Non-Hispanic Black, and 4.2% were Asian/Pacific Islander. Among patients without indication of homelessness/housing instability, 45.3% were Non-Hispanic White, 30.9% were Hispanic, 11.3% were Non-Hispanic Black, and 10.8% were Asian/Pacific Islander. Reflecting in large parts the residential pattern of KPSC membership the majority of seriously ill patients with an indication of homeless/housing instability were located in Los Angeles County (61.3%), followed by San Diego County (14.7%), Orange County (9.2%), and Riverside County (8.4%).

**Table 2.** Characteristics of the study populations and emergency visits within 1 year before/after the index date by the status of homeless/housing instability.

Characteristics	Status of homeless/housing instability			Odd ratio and 95% confidence interval
	No	Yes	Total	
Total (*n*)	205 256	1737	206 993	
Gender*, n* (%)				
Female	107 596 (52.4)	850 (48.9)	108 446 (52.4)	Reference
Male	97 656 (47.6)	887 (51.1)	98 543 (47.6)	1.15 (1.05-1.26)
Other	4 (0.0)	0 (0.0)	4 (0.0)	NE
Race/ethnicity, *n* (col%)				
Non-Hispanic White	92 986 (45.3)	771 (44.4)	93 757 (45.3)	Reference
Hispanic	63 347 (30.9)	540 (31.1)	63 887 (30.9)	1.03 (0.92-1.15)
Non-Hispanic Black	23 178 (11.3)	324 (18.7)	23 502 (11.4)	1.69 (1.48-1.92)
Asian/Pacific Islander	22 152 (10.8)	73 (4.2)	22 225 (10.7)	0.40 (0.32-0.51)
Other/unknown	3593 (1.8)	29 (1.6)	3622 (1.8)	0.92 (0.62, 1.35)
Age (years)				
Mean (SD)	65.9 (15.20)	60.6 (14.81)	65.9 (15.20)	
Median [Q1, Q3]	68.0 (57.0, 77.0)	61.0 (52.0, 71.0)	68.0 (57.0, 77.0)	
Age group (years), *n* (col%)				
18-34	8786 (4.3)	103 (5.9)	8889 (4.3)	Reference
35-44	11 052 (5.4)	140 (8.1)	11 192 (5.4)	1.08 (0.84-1.39)
45-64	64 657 (31.5)	777 (44.7)	65 434 (31.6)	1.02 (0.83-1.26)
65-74	58 177 (28.3)	427 (24.6)	58 604 (28.3)	0.62 (0.50-0.77)
≥75	62 584 (30.5)	290 (16.7)	62 874 (30.4)	0.39 (0.32-0.49)
Emergency visit utilization				
Mean (SD)	0.7 (1.67)	3.0 (8.02)	0.8 (1.83)	
Median [Q1, Q3]	0.0 (0.0, 1.0)	1.0 (0.0, 4.0)	0.0 (0.0, 1.0)	
Emergency visit utilization group *n*, (col%)				
0	135 305 (65.9)	594 (34.2)	135 899 (65.7)	Reference
1-3	59 750 (29.1)	708 (40.8)	60 458 (29.2)	2.70 (2.42-3.0)
4-6	7555 (3.7)	235 (13.5)	7790 (3.8)	7.09 (6.09-8.26)
7-9	1735 (0.8)	91 (5.2)	1826 (0.9)	12.00 (9.58-15.03)
10+	911 (0.4)	109 (6.3)	1020 (0.5)	27.34 (22.07-33.87)
County service area, *n* (col%) ^a^				
Los Angeles	122 594 (59.7)	1064 (61.3)	123 658 (59.7)	Reference
San Diego	28 910 (14.1)	256 (14.7)	29 166 (14.1)	1.02 (0.89-1.17)
Orange County	23 698 (11.5)	159 (9.2)	23 857 (11.5)	0.78 (0.66-0.92)
Riverside	21 675 (10.6)	146 (8.4)	21 821 (10.5)	0.78 (0.66-0.93)
Kern County	4885 (2.4)	29 (1.7)	4914 (2.4)	0.70 (0.48-1.01)
West Ventura County	3143 (1.5)	38 (2.2)	3181 (1.5)	1.41 (1.02-1.95)
Unknown	351 (0.2)	45 (2.6)	396 (0.2)	14.91 (10.88-20.43)

a San Bernardino was combined into Los Angeles and Riverside service area. NE, not estimated.

ED utilization within 1 year of the index date was significantly different between patients with and without indication of homeless/housing instability. The mean number of ED visits was 3.0 among patients identified as homeless/experiencing housing instability compared to 0.7 among those who were not identified as homeless/experiencing housing instability. Importantly, patients with an indication of homelessness/housing were 5 times more likely to have 4 or more ED than patients with no indication of homeless/housing instability.

## Discussions

Addressing social risks and needs is one of the key objectives of Healthy People 2030. ^24^ In this study, we successfully tested and improved an NLP application to identify patients who are homeless/experiencing housing instability among SI patients within the KPSC healthcare system. While the proportion of patients with SI who are also homeless/experiencing housing instability was low (0.84%), a total of 1737 patients were identified to be both seriously ill and homeless/experiencing housing instability. The rate of homeless/at risk of homelessness among the KPSC SI population was nearly 4.7-folded than those in the general population of the United States. ^25^ These patients were significantly more likely to require ED visits and to be high-level users of the ED defined as 4 or more ED visits.

The use of NLP to extract social needs has previously encountered great challenges. ^15–19^ Most current NLP systems are designed to retrieve clinical information by leveraging the standard common terminologies for clinical documents. ^12–13^ The information related to social needs is, however, often not explicitly stated in the clinical notes. Instead, such information needs to be inferred from the care provider’s comments regarding a person’s living situation or environment. Due to the description complexity and variety of social needs in the clinical notes, the performance of NLP applications targeting social needs has historically encountered performance issues. ^15–19^ Our results provide further evidence of the difficulty of generating a highly generalizable NLP algorithm for social needs. Description of homelessness may vary by care setting (eg, ED, specialist, mental health visit), the population under study (a general population of patients seen in the ED vs a highly medically vulnerable population who is seen frequently by medical specialists), and individual health provider’s practices. The initial KPSC algorithm as published in Hatef et al ^19^ had a high PPV of 100%. When we applied the same algorithm to the cohort of seriously ill patients it resulted in a reduced PPV (60%) compared to its performance in our prior study. We used chart review to identify patterns that were either different or more common for the seriously ill population. As shown in Table S2, the current study led us to drop 32 patterns and added 50 new patterns. Our experience suggests that the development of other NLP algorithms for social needs will likely require large samples for training to capture various lemma variants and linguistic patterns and should be tested on various populations and healthcare settings.

Our study acknowledges several important limitations. First, our algorithm relied on the available information and the accuracy of the EHRs. Clinical notes are not available for individuals seeking care at non-KPSC facilities which may be particularly true for patients experiencing homelessness. Therefore, our estimation of the prevalence of homeless/housing instability among patients with SI may be underestimating the magnitude of the problem. Second, although the algorithm identified homelessness/housing instability at the sentence and note level and then aggregated the information to the patient level, the validation of the algorithm with chart review was only conducted at the patient level. Further evaluation of instance- or note-level performance can strengthen the robustness of the algorithm. Finally, our study only provides initial evidence for the clinical usefulness of our NLP algorithm by demonstrating a higher rate of health care usage of patients at risk of homelessness/homeless. Further examining the characteristics of patients who are at risk of homelessness/homelessness and the timing of homelessness in respect to the patients’ SI diagnoses warrant further in-depth analysis that we hope to perform in the future.

In conclusion, the improved NLP algorithm effectively identified patients with SI who are homeless or are experiencing housing instability. We also showed that these patients had to rely disproportionally on the ED to manage their SI. We hope that providing a systematic approach to identifying these patients who are both socially and medically highly vulnerable provides the first step to designing and implementing systemic interventions for this small but highly relevant population.

## Supplementary Material

ooad082_Supplementary_Data

## Data Availability

The data underlying this article were extracted from the electronic health records and cannot be shared publicly for the privacy of individuals that participated in the study.
