# Supplementary material for: Using natural language processing to identify the status of homelessness and housing instability among serious illness patients from clinical notes in an integrated healthcare system
Source: JAMIA Open. 2023 Sep 22;6(3):ooad082. doi: 10.1093/jamiaopen/ooad082 (PMC10517738; doi:10.1093/jamiaopen/ooad082)
Supplement: ooad082_Supplementary_Data [file ooad082_supplementary_data.docx]

Table S1. Homeless/housing insecurity related keyword or phrases.

| Category | Keyword or Phrases* |
| --- | --- |
| Homelessness | homelessness, homeless, houseless, unhoused, shelter, hostel, motel, housing, lose (home\|house\|housing), (no\|don’t have\|without) (place\|home\|house\|housing) to (live\|stay\|reside\|sleep), (live\|stay\|reside\|sleep\|wander) (on\|at\|in) (hostel\|motel\|shelter\|street\|park\|parking lot\|bus station\|bus stop\|train station\|train stop\|terminal\|vehicle\|car\|jeep\|van\|boat), (return\|back) to (hostel\|motel\|shelter\|street), discharge to (hostel\|motel\|shelter), temporal (stay\|live) with (friend\|relative), lahsa, hopics, coordinated entry system, (not\|don’t have\|lack\|without) (nighttime\|permanent\|stable\|steady) place to (stay\|sleep\|live\|reside), (no\|don’t have\|lack\|without) (nighttime\|permanent\|steady\|stable) (residence\|address\|housing\|house\|home\|place), kick out (of\|from) (home\|house), has no (home\|address), no where to place patient, no where to (live\|stay\|reside\|sleep), want help find (place\|house\|home\|shelter) to (live\|stay\|reside\|sleep) |
| Housing insecurity | housing (insecurity\|instability\|housing subsidy), evicted, eviction note, (can’t\|unable to\|not able to\|hard\|difficulty\|trouble\|worry\|concern) (pay\|afford) (rent\|mortgage\|apartment\|housing\|home), delinquent (loan\| mortgage), apply for housing, (unstable\|unsteady\|unreliable) housing, unstable living situation, (receive\|obtain\|need\|require\|ask\|get) housing (help\|support\|assistance), (house\|apartment\|home) (occupy\|rent) without rent payment |

* Plural, verbs and abbreviations are also included for searching

(..|..) indicates on any word or terms between “|” within the paretheses.

Table S2. Summary of the spaCy homelessness and housing instability phrase patterns used for matching in previous study and current study.

| Study | Phrase pattern |
| --- | --- |
| both | "label": "HOMELESSNESS", "pattern": ["LEMMA": "apply", "POS": "ADP", "OP": "?", "LEMMA": "IN": ["housing", "shelter"]] |
| both | "label": "HOMELESSNESS", "pattern": ["LEMMA": "barrow", "OP": "?", "LEMMA": "couch", "POS": "ADP", "OP": "?", "LEMMA": "friend", "OP": "?", "LEMMA": "sleep"] |
| both | "label": "HOMELESSNESS", "pattern": ["LEMMA": "barrow", "OP": "?", "LEMMA": "friend", "OP": "?", "LEMMA": "couch", "OP": "?", "LEMMA": "sleep"] |
| both | "label": "HOMELESSNESS", "pattern": ["LEMMA": "find", "OP": "?", "OP": "?", "LOWER": "local", "OP": "?", "LEMMA": "shelter"] |
| both | "label": "HOMELESSNESS", "pattern": ["LEMMA": "give", "OP": "?", "OP": "?", "LOWER": "list", "OP": "?", "LEMMA": "shelter"] |
| both | "label": "HOMELESSNESS", "pattern": ["LEMMA": "temporarily","LEMMA": "IN": ["stay", "live"],"OP": "?","OP": "?","POS": "ADP","OP": "?","OP": "?","LEMMA": "IN": ["friend", "relative"]] |
| both | "label": "HOMELESSNESS", "pattern": ["LEMMA": "undercare", "LOWER": "of", "LOWER": "homeless", "LEMMA": "advocacy"] |
| both | "label": "HOMELESSNESS", "pattern": ["LEMMA": "IN": ["no", "not"], "OP": "?", "LEMMA": "have", "LEMMA": "nighttime", "OP": "?","LEMMA": "residence"] |
| both | "label": "HOMELESSNESS", "pattern": ["LEMMA": "IN": ["no", "not"], "OP": "?", "LEMMA": "have", "LEMMA": "IN": ["nighttime", "permanent", "steady", "stable"], "LEMMA": "place", "LEMMA": "to", "LEMMA": "IN": ["stay", "sleep", "live","reside","wander"]] |
| both | "label": "HOMELESSNESS", "pattern": ["LEMMA": "IN": ["no", "not"], "OP": "?", "LEMMA": "have", "OP": "?", "OP": "?", "LEMMA": "IN": ["nighttime", "permanent", "steady", "stable"], "OP": "?","LEMMA": "IN": ["residence", "housing", "address"]] |
| both | "label": "HOMELESSNESS", "pattern": ["LEMMA": "IN": ["pt","pat","patient"],"POS": "AUX","OP": "?","OP": "?","LEMMA": "shelter"] |
| both | "label": "HOMELESSNESS", "pattern": ["LEMMA": "IN": ["sleep", "stay", "live", "move","reside","wander"],"OP": "?","OP": "?","POS": "ADP","OP": "?","LEMMA": "low","LEMMA": "income","LEMMA": "IN": ["shelter", "hostel", "motel", "housing"]] |
| both | "label": "HOMELESSNESS", "pattern": ["LEMMA": "IN": ["sleep", "stay", "live","reside","wander"],"LOWER":"IN":["in","to","on","at"],"OP": "?","OP": "?","LEMMA": "IN": ["bus","train"],"LEMMA": "IN": ["station", "stop", "terminal"]] |
| both | "label": "HOMELESSNESS", "pattern": ["LOWER": "evicted"] |
| both | "label": "HOMELESSNESS", "pattern": ["LOWER": "lack", "OP": "?", "LEMMA": "IN": ["nighttime", "permanent", "steady", "stable"] ,"LEMMA": "IN": ["housing","house","home"]] |
| both | "label": "HOMELESSNESS", "pattern": ["LOWER":"IN":["pt","pat","patient","he","she","i"], "OP": "?", "OP": "?", "OP": "?", "LEMMA": "kick","OP": "?", "OP": "?","OP": "?", "OP": "?","OP": "?","LOWER": "IN": ["of","from"],"OP": "?","LEMMA":"IN": ["home","house"]] |
| both | "label": "HOMELESSNESS", "pattern": ["POS": "AUX","OP": "?","OP": "?","LEMMA": "shelter", "LEMMA": "wait", "LEMMA": "list"] |
| both | "label": "HOUSING_INSTABILITY", "pattern": ["LEMMA": "apply", "POS": "ADP", "OP": "?", "LEMMA": "housing"] |
| both | "label": "HOUSING_INSTABILITY", "pattern": ["LEMMA": "assess", "OP": "?", "LEMMA": "housing", "LEMMA": "need"] |
| both | "label": "HOUSING_INSTABILITY", "pattern": ["LEMMA": "assign", "OP": "?", "LEMMA": "case", "LEMMA": "manager","POS": "ADP", "OP": "?", "LEMMA": "housing"] |
| both | "label": "HOUSING_INSTABILITY", "pattern": ["LEMMA": "assist", "OP": "?", "LEMMA": "housing", "LEMMA": "benefit"] |
| both | "label": "HOUSING_INSTABILITY", "pattern": ["LEMMA": "can", "LEMMA": "not", "OP": "?", "LEMMA": "obtain", "OP": "?", "OP": "?", "LEMMA": "housing"] |
| both | "label": "HOUSING_INSTABILITY", "pattern": ["LEMMA": "coordinate", "OP": "?", "LEMMA": "housing", "LEMMA": "service"] |
| both | "label": "HOUSING_INSTABILITY", "pattern": ["LEMMA": "delinquent", "LEMMA": "IN": ["loan", "mortgage"]] |
| both | "label": "HOUSING_INSTABILITY", "pattern": ["LEMMA": "fill", "LOWER": "out", "OP": "?", "LEMMA": "housing", "LEMMA": "application"] |
| both | "label": "HOUSING_INSTABILITY", "pattern": ["LEMMA": "has", "OP": "?", "LOWER": "no", "OP": "?", "OP": "?", "LEMMA": "IN": ["home", "house", "address"]] |
| both | "label": "HOUSING_INSTABILITY", "pattern": ["LEMMA": "housing", "LEMMA": "IN": ["instability","subsidy","insecurity"]] |
| both | "label": "HOUSING_INSTABILITY", "pattern": ["LEMMA": "IN": ["house", "apartment", "home", "place"], "OP": "?", "OP": "?", "LEMMA": "IN": ["occupy", "rent"], "OP": "?", "OP": "?", "OP": "?", "LOWER": "without", "OP": "?", "LOWER": "rent", "LOWER": "payment"] |
| both | "label": "HOUSING_INSTABILITY", "pattern": ["LEMMA": "IN": ["receive", "obtain", "need", "require", "ask", "get"], "OP": "?", "LEMMA": "IN": ["help", "support", "assistance"], "OP": "?", "LEMMA": "housing"] |
| both | "label": "HOUSING_INSTABILITY", "pattern": ["LEMMA": "IN": ["refer", "recommend"], "LEMMA": "to","OP": "?","OP": "?" ,"LOWER": "housing"] |
| both | "label": "HOUSING_INSTABILITY", "pattern": ["LEMMA": "IN": ["request","ask","look","seek"],"LEMMA": "information","OP":"?","OP":"?","LEMMA": "shelter"] |
| both | "label": "HOUSING_INSTABILITY", "pattern": ["LEMMA": "IN": ["request","ask","look","seek"],"LOWER": "for","OP":"?","OP":"?","OP":"?","OP":"?","OP":"?","LEMMA": "shelter"] |
| both | "label": "HOUSING_INSTABILITY", "pattern": ["LEMMA": "IN": ["sleep", "stay", "live", "move","reside","wander"],"LOWER": "IN": ["in","at","to"],"OP":"?","LEMMA": "motor","LEMMA":"home"] |
| both | "label": "HOUSING_INSTABILITY", "pattern": ["LEMMA": "IN": ["sleep", "stay", "live", "move","reside","wander"],"OP": "?","OP": "?","POS": "ADP","OP": "?","LEMMA": "IN": ["inadequate", "unsatisfactory", "inappropriate"],"OP": "?","LEMMA": "IN": ["housing", "house", "apartment", "home", "place"]] |
| both | "label": "HOUSING_INSTABILITY", "pattern": ["LEMMA": "IN": ["unstable", "unsteady", "unreliable"], "OP": "?", "LEMMA": "housing"] |
| both | "label": "HOUSING_INSTABILITY", "pattern": ["LOWER": "given", "LEMMA": "eviction", "OP": "?", "LEMMA": "notice"] |
| both | "label": "HOUSING_INSTABILITY", "pattern": ["LOWER": "need", "OP": "?","OP": "?" ,"LOWER": "housing"] |
| both | "label": "HOUSING_INSTABILITY", "pattern":["LOWER":"axis","LOWER":"IN":["v","iv"],"OP":"?","LEMMA":"housing","LEMMA":"problem"] |
| both | "label":"HOMELESSNESS","pattern":["LEMMA":"arrange","OP":"?","LEMMA":"housing","OP":"?","OP":"?","OP":"?","LEMMA":"IN":["motel","inn"]] |
| both | "label":"HOMELESSNESS","pattern":["LEMMA":"be","LOWER":"evicted"] |
| both | "label":"HOMELESSNESS","pattern":["LEMMA":"change","OP":"?","LEMMA":"mind","LOWER":"on","LEMMA":"IN":["refuse","refusal"],"OP":"?","LEMMA":"shelter"] |
| both | "label":"HOMELESSNESS","pattern":["LEMMA":"diagnosis","OP":"?","LEMMA":"IN":["homeless","houseless","unhoused"]] |
| both | "label":"HOMELESSNESS","pattern":["LEMMA":"discharge","LEMMA":"IN":["destination","plan"],"OP":"?","OP":"?","LEMMA":"shelter"] |
| both | "label":"HOMELESSNESS","pattern":["LEMMA":"discharge","LOWER":"IN":["pt","pat","patient","him","her"],"LOWER":"to","OP":"?","OP":"?","LEMMA":"IN":["shelter","street","motel","hostel"]] |
| both | "label":"HOMELESSNESS","pattern":["LEMMA":"discharge","OP":"?","OP":"?","LEMMA":"path","LOWER":"of","LEMMA":"life","LEMMA":"shelter"] |
| both | "label":"HOMELESSNESS","pattern":["LEMMA":"discharge","OP":"?","OP":"?","LOWER":"per","LEMMA":"homeless","LEMMA":"IN":["protocol","policy"]] |
| both | "label":"HOMELESSNESS","pattern":["LEMMA":"discharge","OP":"?","OP":"?","OP":"?","OP":"?","OP":"?","LOWER":"per","OP":"?","LEMMA":"homeless","LEMMA":"IN":["protocol","policy"]] |
| both | "label":"HOMELESSNESS","pattern":["LEMMA":"endorse","OP":"?","OP":"?","LEMMA":"shelter"] |
| both | "label":"HOMELESSNESS","pattern":["LEMMA":"financial","LEMMA":"IN":["issue","stress"],"LEMMA":"IN":["homeless","houseless","unhoused"]] |
| both | "label":"HOMELESSNESS","pattern":["LEMMA":"help","OP":"?","LOWER":"IN":["pt","pat","patient","him","her"],"OP":"?","OP":"?","OP":"?","OP":"?","OP":"?","LEMMA":"find","OP":"?","OP":"?","LEMMA":"IN":["housing","shelter"]] |
| both | "label":"HOMELESSNESS","pattern":["LEMMA":"homeless","LEMMA":"log","OP":"?","LOWER":"completed"] |
| both | "label":"HOMELESSNESS","pattern":["LEMMA":"homeless","LEMMA":"present","OP":"?","OP":"?","LOWER":"IN":["ed","emergency"]] |
| both | "label":"HOMELESSNESS","pattern":["LEMMA":"homeless","LEMMA":"protocol","OP":"?","LOWER":"initiated"] |
| both | "label":"HOMELESSNESS","pattern":["LEMMA":"homeless","LEMMA":"protocol","OP":"?","OP":"?","LOWER":"IN":["followed","done","completed"]] |
| both | "label":"HOMELESSNESS","pattern":["LEMMA":"homeless","LEMMA":"summary","LEMMA":"note"] |
| both | "label":"HOMELESSNESS","pattern":["LEMMA":"homeless","LEMMA":"IN":["resource","shelter"],"OP":"?","OP":"?","OP":"?","OP":"?","OP":"?","LOWER":"IN":["provided","given"]] |
| both | "label":"HOMELESSNESS","pattern":["LEMMA":"homeless","LOWER":"dc","LEMMA":"checklist"] |
| both | "label":"HOMELESSNESS","pattern":["LEMMA":"homelessness"] |
| both | "label":"HOMELESSNESS","pattern":["LEMMA":"identify","LOWER":"as","LEMMA":"IN":["homeless","houseless","unhoused"]] |
| both | "label":"HOMELESSNESS","pattern":["LEMMA":"leave","OP":"?","OP":"?","LOWER":"with","OP":"?","OP":"?","LOWER":"to","OP":"?","OP":"?","LEMMA":"shelter"] |
| both | "label":"HOMELESSNESS","pattern":["LEMMA":"live","LOWER":"with","OP":"?","OP":"?","LEMMA":"IN":["homeless","houseless","unhoused"]] |
| both | "label":"HOMELESSNESS","pattern":["LEMMA":"live","LOWER":"IN":["in","at"],"OP":"?","LEMMA":"apartment","LOWER":"for","LEMMA":"IN":["homeless","houseless","unhoused"]] |
| both | "label":"HOMELESSNESS","pattern":["LEMMA":"medical","LEMMA":"social","LEMMA":"work","LEMMA":"homeless","LEMMA":"summary"] |
| both | "label":"HOMELESSNESS","pattern":["LEMMA":"path","LOWER":"of","LEMMA":"life","LEMMA":"shelter","LEMMA":"current","LEMMA":"placement"] |
| both | "label":"HOMELESSNESS","pattern":["LEMMA":"plan","OP":"?","LEMMA":"connect","OP":"?","LEMMA":"housing","LEMMA":"opportunity"] |
| both | "label":"HOMELESSNESS","pattern":["LEMMA":"reason","LOWER":"for","OP":"?","LEMMA":"IN":["homeless","houseless","unhoused"]] |
| both | "label":"HOMELESSNESS","pattern":["LEMMA":"reason","OP":"?","OP":"?","OP":"?","OP":"?","OP":"?","LOWER":"IN":["consult","consults","consultation","consultations"],"LEMMA":"IN":["homeless","houseless","unhoused"],"LEMMA":"barrier"] |
| both | "label":"HOMELESSNESS","pattern":["LEMMA":"referral","LEMMA":"IN":["homeless","houseless","unhoused","housing"],"LEMMA":"shelter"] |
| both | "label":"HOMELESSNESS","pattern":["LEMMA":"render","OP":"?","OP":"?","LEMMA":"IN":["homeless","houseless","unhoused"]] |
| both | "label":"HOMELESSNESS","pattern":["LEMMA":"transfer","LOWER":"to","OP":"?","OP":"?","LEMMA":"shelter"] |
| both | "label":"HOMELESSNESS","pattern":["LEMMA":"transportation","OP":"?","OP":"?","LOWER":"provided","OP":"?","OP":"?","OP":"?","LOWER":"per","LEMMA":"homeless","LEMMA":"IN":["protocol","policy"]] |
| both | "label":"HOMELESSNESS","pattern":["LEMMA":"year","LOWER":"old","LEMMA":"homeless"] |
| both | "label":"HOMELESSNESS","pattern":["LEMMA":"year","LOWER":"old","LOWER":"IN":["male","female","woman","man"],"LEMMA":"homeless"] |
| both | "label":"HOMELESSNESS","pattern":["LEMMA":"IN":["accept","sign","signed"],"OP":"?","LEMMA":"shelter"] |
| both | "label":"HOMELESSNESS","pattern":["LEMMA":"IN":["assessment","problem","status","msw"],"OP":"?","OP":"?","LEMMA":"IN":["homeless","houseless","unhoused"]] |
| both | "label":"HOMELESSNESS","pattern":["LEMMA":"IN":["give","provide","educate","connect"],"OP":"?","OP":"?","OP":"?","OP":"?","LEMMA":"IN":["homeless","shelter"],"LEMMA":"IN":["resource","option"]] |
| both | "label":"HOMELESSNESS","pattern":["LEMMA":"IN":["give","provide","inform","educate"],"OP":"?","LOWER":"IN":["pt","pat","patient","him","her"],"OP":"?","OP":"?","OP":"?","OP":"?","OP":"?","LEMMA":"IN":["resource","information"],"OP":"?","OP":"?","OP":"?","OP":"?","OP":"?","LEMMA":"IN":["homeless","shelter"]] |
| both | "label":"HOMELESSNESS","pattern":["LEMMA":"IN":["give","provide"],"OP":"?","OP":"?","LEMMA":"list","OP":"?","OP":"?","LEMMA":"shelter"] |
| both | "label":"HOMELESSNESS","pattern":["LEMMA":"IN":["homeless","houseless","unhoused","shelter"],"LEMMA":"IN":["log","packet","form","package"],"OP":"?","OP":"?","LOWER":"IN":["initiated","done","completed","complete","finished","signed"]] |
| both | "label":"HOMELESSNESS","pattern":["LEMMA":"IN":["homeless","houseless","unhoused"],"LOWER":"for","OP":"?","OP":"?","OP":"?","LEMMA":"IN":["year","month","week","day","yr","yrs","mon","mons"]] |
| both | "label":"HOMELESSNESS","pattern":["LEMMA":"IN":["homeless","houseless","unhoused"],"LOWER":"IN":["pt","pat","patient","since"]] |
| both | "label":"HOMELESSNESS","pattern":["LEMMA":"IN":["homeless","houseless","unhoused"],"LOWER":"IN":["unemployed","confirmed"]] |
| both | "label":"HOMELESSNESS","pattern":["LEMMA":"IN":["homeless","houseless","unhoused"],"OP":"?","LEMMA":"IN":["assessment","problem","summary"]] |
| both | "label":"HOMELESSNESS","pattern":["LEMMA":"IN":["homeless","houseless","unhoused"],"OP":"?","OP":"?","LEMMA":"shelter","LEMMA":"IN":["checklist","list"]] |
| both | "label":"HOMELESSNESS","pattern":["LEMMA":"IN":["homeless","houseless","unhoused"],"OP":"?","OP":"?","LOWER":"seen","LOWER":"by"] |
| both | "label":"HOMELESSNESS","pattern":["LEMMA":"IN":["homeless","shelter"],"LEMMA":"IN":["discharge","placement","huddle"]] |
| both | "label":"HOMELESSNESS","pattern":["LEMMA":"IN":["homeless","shelter"],"OP":"?","OP":"?","LOWER":"provided"] |
| both | "label":"HOMELESSNESS","pattern":["LEMMA":"IN":["provide","inform","educate"],"OP":"?","LOWER":"IN":["pt","pat","patient","him","her"],"OP":"?","OP":"?","OP":"?","OP":"?","OP":"?","LEMMA":"shelter","OP":"?","OP":"?","LEMMA":"work","LEMMA":"on","LEMMA":"first","LEMMA":"come","LEMMA":"first","LEMMA":"serve","LEMMA":"basis"] |
| both | "label":"HOMELESSNESS","pattern":["LEMMA":"IN":["remain","become"],"OP":"?","LEMMA":"IN":["homeless","houseless","unhoused"]] |
| both | "label":"HOMELESSNESS","pattern":["LEMMA":"IN":["shelter","homeless"],"OP":"?","OP":"?","LEMMA":"paper","LEMMA":"work","OP":"?","OP":"?","LOWER":"IN":["signed","completed"]] |
| both | "label":"HOMELESSNESS","pattern":["LEMMA":"IN":["shelter","homeless"],"OP":"?","OP":"?","LEMMA":"IN":["checklist","paperwork","paper","packet","protocol"],"OP":"?","OP":"?","LOWER":"IN":["signed","completed","done","placed"]] |
| both | "label":"HOMELESSNESS","pattern":["LEMMA":"IN":["shelter","homeless"],"OP":"?","OP":"?","LEMMA":"IN":["list","checklist","paperwork","paper","protocol"],"OP":"?","OP":"?","LOWER":"IN":["placed","completed","done"]] |
| both | "label":"HOMELESSNESS","pattern":["LEMMA":"IN":["sign","complete"],"OP":"?","LEMMA":"IN":["shelter","homeless"],"OP":"?","LEMMA":"paper","LEMMA":"work"] |
| both | "label":"HOMELESSNESS","pattern":["LEMMA":"IN":["social","issue"],"LEMMA":"IN":["homeless","houseless","unhoused"]] |
| both | "label":"HOMELESSNESS","pattern":["LEMMA":"IN":["want","accept","wish","pend","choose","place"],"OP":"?","OP":"?","LEMMA":"shelter"] |
| both | "label":"HOMELESSNESS","pattern":["LOWER": "per", "OP": "?", "OP": "?", "LEMMA": "homeless", "LEMMA": "policy", "LOWER":"IN":["pt","pat","patient"],"LEMMA": "be","LOWER": "provided"] |
| both | "label":"HOMELESSNESS","pattern":["LOWER":"axis","LOWER":"IN":["v","iv"],"OP":"?","LEMMA":"homeless","LEMMA":"problem"] |
| both | "label":"HOMELESSNESS","pattern":["LOWER":"being","LEMMA":"IN":["homeless","houseless","unhoused"]] |
| both | "label":"HOMELESSNESS","pattern":["LOWER":"living","LEMMA":"IN":["arrangement","situation"],"OP":"?","OP":"?","OP":"?","LEMMA":"IN":["homeless","houseless","unhoused"]] |
| both | "label":"HOMELESSNESS","pattern":["LOWER":"living","LOWER":"situation","LOWER":"who","LOWER":"lives","LOWER":"in","LOWER":"the","LOWER":"home","LOWER":"stable","LOWER":"housing","LEMMA":"IN":["homeless","houseless","unhoused"]] |
| both | "label":"HOMELESSNESS","pattern":["LOWER":"long","LOWER":"term","LEMMA":"plan","OP":"?","LEMMA":"shelter"] |
| both | "label":"HOMELESSNESS","pattern":["LOWER":"no","LEMMA":"address","OP":"?","LEMMA":"IN":["homeless","houseless","unhoused"]] |
| both | "label":"HOMELESSNESS","pattern":["LOWER":"no","LEMMA":"where","LOWER":"to","LOWER":"place","LOWER":"IN":["pt","pat","patient","him","her"]] |
| both | "label":"HOMELESSNESS","pattern":["LOWER":"poor","LOWER":"living","LEMMA":"condition","OP":"?","OP":"?","LEMMA":"IN":["homeless","houseless","unhoused"]] |
| both | "label":"HOMELESSNESS","pattern":["LOWER":"yo","LEMMA":"homeless"] |
| both | "label":"HOMELESSNESS","pattern":["LOWER":"yo","LOWER":"IN":["male","female","woman","man"],"LEMMA":"homeless"] |
| both | "label":"HOMELESSNESS","pattern":["LOWER":"IN":["current","currently"],"OP":"?","LEMMA":"IN":["homeless","houseless","unhoused"]] |
| both | "label":"HOMELESSNESS","pattern":["LOWER":"IN":["due","d"],"LOWER":"IN":["to","t"],"LEMMA":"homeless","LEMMA":"IN":["status","issue"]] |
| both | "label":"HOMELESSNESS","pattern":["LOWER":"IN":["due","d"],"LOWER":"IN":["to","t"],"LEMMA":"housing","LEMMA":"IN":["insecurity","issue","instability"]] |
| both | "label":"HOMELESSNESS","pattern":["LOWER":"IN":["have","has"],"LOWER":"been","OP":"?","OP":"?","LEMMA":"IN":["homeless","houseless","unhoused"]] |
| both | "label":"HOMELESSNESS","pattern":["LOWER":"IN":["initiated","done","completed","complete","finished","sign","signed"],"OP":"?","OP":"?","OP":"?","LEMMA":"IN":["homeless","houseless","unhoused","shelter"],"LEMMA":"IN":["log","packet","form","package"]] |
| both | "label":"HOMELESSNESS","pattern":["LOWER":"IN":["pt","pat","patient","he","she","i"],"LOWER":"has","LOWER":"seen","OP":"?","OP":"?","LEMMA":"shelter","LOWER":"in"] |
| both | "label":"HOMELESSNESS","pattern":["LOWER":"IN":["pt","pat","patient","he","she","i"],"LOWER":"has","OP":"?","OP":"?","LOWER":"place","LOWER":"for","LEMMA":"discharge"] |
| both | "label":"HOMELESSNESS","pattern":["LOWER":"IN":["pt","pat","patient","he","she","i"],"LOWER":"IN":["do","does"],"LOWER":"not","LOWER":"have","OP":"?","OP":"?","LOWER":"IN":["place","places"],"LOWER":"to","LOWER":"IN":["live","stay"]] |
| both | "label":"HOMELESSNESS","pattern":["LOWER":"IN":["pt","pat","patient","he","she","i"],"LOWER":"IN":["don","doesn"],"LOWER":"t","LOWER":"have","OP":"?","OP":"?","LOWER":"IN":["place","places"],"LOWER":"to","LOWER":"IN":["live","stay"]] |
| both | "label":"HOMELESSNESS","pattern":["LOWER":"IN":["pt","pat","patient","he","she","i"],"LOWER":"IN":["dont","doesnt"],"LOWER":"have","OP":"?","OP":"?","LOWER":"IN":["place","places"],"LOWER":"to","LOWER":"IN":["live","stay"]] |
| both | "label":"HOMELESSNESS","pattern":["LOWER":"IN":["pt","pat","patient","he","she","i"],"LOWER":"IN":["has","have"],"OP":"?","LEMMA":"IN":["place","placement"],"LOWER":"IN":["in","at"],"OP":"?","LEMMA":"shelter"] |
| both | "label":"HOMELESSNESS","pattern":["LOWER":"IN":["pt","pat","patient","he","she","i"],"LOWER":"IN":["is","was","be","being","am"],"OP":"?","OP":"?","LEMMA":"IN":["homeless","houseless","unhoused"],"LEMMA":"IN":["female","male","woman","man"]] |
| both | "label":"HOMELESSNESS","pattern":["LOWER":"IN":["pt","pat","patient","he","she","i"],"OP":"?","OP":"?","LEMMA":"discharge","OP":"?","OP":"?","OP":"?","OP":"?","OP":"?","LEMMA":"homeless","LEMMA":"IN":["policy","protocol"]] |
| both | "label":"HOMELESSNESS","pattern":["LOWER":"IN":["pt","pat","patient","he","she","i"],"OP":"?","OP":"?","LEMMA":"IN":["homeless","houseless","unhoused"]] |
| both | "label":"HOMELESSNESS","pattern":["LOWER":"IN":["pt","pat","patient","he","she","i"],"OP":"?","OP":"?","LOWER":"discharged","OP":"?","OP":"?","OP":"?","OP":"?","OP":"?","LEMMA":"homeless","LEMMA":"IN":["policy","protocol"]] |
| both | "label":"HOMELESSNESS","pattern":["LOWER":"IN":["pt","pat","patient","he","she","i"],"OP":"?","OP":"?","LOWER":"evicted"] |
| both | "label":"HOMELESSNESS","pattern":["LOWER":"IN":["pt","pat","patient","he","she"],"OP":"?","LEMMA": "IN": ["look","seek"],"LOWER":"for","OP":"?","LEMMA":"break","LOWER":"IN":["from","at"],"OP":"?","LEMMA": "IN": ["street","shelter"]] |
| both | "label":"HOMELESSNESS","pattern":["LOWER":"IN":["pt","pat","patient","he","she"],"OP":"?","LEMMA":"choose","OP":"?","OP":"?","LEMMA":"shelter"] |
| both | "label":"HOMELESSNESS","pattern":["LOWER":"IN":["pt","pat","patient","her","his"],"OP":"?","LEMMA":"address","LEMMA":"be","OP":"?","OP":"?","LEMMA":"IN":["homeless","houseless","unhoused"]] |
| both | "label":"HOMELESSNESS","pattern":["LOWER":"IN":["pt","pat","patient"],"OP": "?", "OP": "?", "OP": "?", "OP": "?", "OP": "?","LEMMA": "issue","LOWER": "with", "LEMMA":"IN":["homeless","houseless","unhoused"]] |
| both | "label":"HOMELESSNESS","pattern":["LOWER":"IN":["unemployed","confirmed"],"LEMMA":"IN":["homeless","houseless","unhoused"]] |
| both | "label":"HOUSING_INSTABILITY","pattern":["LEMMA":"evaluate","OP":"?","OP":"?","OP":"?","LEMMA":"housing","LEMMA":"situation"] |
| both | "label":"HOUSING_INSTABILITY","pattern":["LEMMA":"problem","LEMMA":"be","LEMMA":"housing"] |
| both | "label":"HOUSING_INSTABILITY","pattern":["LEMMA":"unstable","LOWER":"IN":["housing","living"],"LEMMA":"situation"] |
| current | "label": "HOMELESSNESS", "pattern": ["LEMMA": "address", "LOWER":"IN": ["unk","unknown"], "LEMMA":"IN": ["homeless","houseless","unhoused"]] |
| current | "label": "HOMELESSNESS", "pattern": ["LEMMA": "has", "OP": "?", "LOWER": "no","LOWER":"IN":[ "where","place"], "OP": "?", "OP": "?", "LEMMA": "IN": ["live"]] |
| current | "label": "HOMELESSNESS", "pattern": ["LEMMA": "has", "OP": "?", "LOWER":"IN":[ "nowhere","noplace"], "OP": "?", "OP": "?", "LEMMA": "IN": ["live"]] |
| current | "label": "HOMELESSNESS", "pattern": ["LEMMA": "no","LEMMA":"IN": ["where","place"], "LEMMA": "to", "LEMMA": "IN": ["live"]] |
| current | "label": "HOMELESSNESS", "pattern": ["LEMMA": "IN": ["housing","house","home"], "LEMMA":"accessibility", "OP": "?", "OP": "?","LEMMA":"IN": ["homeless","houseless","unhoused"]] |
| current | "label": "HOMELESSNESS", "pattern": ["LEMMA": "IN": ["live", "move","reside","wander"],"OP": "?","OP": "?","LOWER":"IN":["in","to","on","at"],"OP": "?","OP": "?","OP": "?","LEMMA": "IN": ["shelter", "hostel", "motel"]] |
| current | "label": "HOMELESSNESS", "pattern": ["LEMMA": "IN": ["live","lives","reside"],"LOWER": "in","OP": "?","LEMMA": "IN": ["car","vehicle","jeep","van"]] |
| current | "label": "HOMELESSNESS", "pattern": ["LEMMA": "IN": ["live","reside"],"LOWER": "out","LOWER":"of","OP": "?","LEMMA": "IN": ["shelter","car","vehicle","jeep","van"]] |
| current | "label": "HOMELESSNESS", "pattern": ["LEMMA": "IN": ["no", "not"], "OP": "?", "LEMMA": "have", "OP": "?", "LEMMA":"IN": ["place","house","home","shelter"], "LEMMA": "to", "LEMMA": "IN": ["sleep", "stay", "live", "reside","wander"]] |
| current | "label": "HOMELESSNESS", "pattern": ["LEMMA":"disposition", "LEMMA":"IN": ["homeless","home"], "LEMMA":"shelter"] |
| current | "label": "HOMELESSNESS", "pattern": ["LEMMA":"identify", "OP": "?", "LOWER":"as", "OP": "?", "LEMMA":"IN": ["homeless","houseless","unhoused"]] |
| current | "label": "HOMELESSNESS", "pattern": ["LEMMA":"want","LEMMA": "help", "OP": "?","LEMMA": "find", "OP": "?", "LEMMA":"IN": ["place","house","home","shelter"], "LEMMA": "to", "LEMMA": "IN": ["sleep", "stay", "live", "reside","wander"]] |
| current | "label": "HOMELESSNESS", "pattern": ["LEMMA":"IN": ["admit","admission"], "LEMMA": "source", "LEMMA":"IN": ["homeless","houseless","unhoused"]] |
| current | "label": "HOMELESSNESS", "pattern": ["LEMMA":"IN": ["homeless","houseless","unhoused"],"OP": "?", "OP": "?","LEMMA":"separate","LOWER":"IN":["from","by"]] |
| current | "label": "HOMELESSNESS", "pattern": ["LEMMA":"IN": ["nowhere","noplace"], "LEMMA": "to", "LEMMA": "IN": ["live"]] |
| current | "label": "HOMELESSNESS", "pattern": ["TEXT":"REGEX":"(pa?t\|patient\|he\|she)", "OP": "?", "TEXT":"REGEX":"accept(s?\|ed\|ing)", "LEMMA": "resource", "LOWER": "for", "LEMMA":"IN": ["homeless","houseless","unhoused"]] |
| current | "label": "HOMELESSNESS", "pattern": ["TEXT":"REGEX":"(pa?t\|patient\|he\|she)", "OP": "?", "TEXT":"REGEX":"accept(s?\|ed\|ing)", "OP": "?", "LEMMA":"IN": ["homeless","houseless","unhoused"], "LEMMA": "resource"] |
| current | "label": "HOMELESSNESS", "pattern": ["TEXT":"REGEX":"financ(e\|ial)", "LEMMA":"issue", "LOWER": "yes", "OP": "?", "OP": "?","LEMMA":"IN": ["homeless","houseless","unhoused"]] |
| current | "label": "HOUSING_INSTABILITY", "pattern": ["LEMMA": "can", "LEMMA": "not", "OP": "?", "LEMMA": "IN": ["pay", "afford"],"LOWER": "IN": ["my","his","her","their","the","our","this","that","one"],"LEMMA": "IN": ["housing", "house", "apartment", "home", "rent", "mortgage"]] |
| current | "label": "HOUSING_INSTABILITY", "pattern": ["LEMMA": "lose","LOWER": "IN": ["the","my","his", "her","their","our","this","that","one"], "LEMMA": "IN": ["home", "house","housing"]] |
| current | "label": "HOUSING_INSTABILITY", "pattern": ["LEMMA": "lose","LOWER": "IN": ["the","my","his", "her","their","our","this","that","one"],"LEMMA": "IN": ["current"], "LEMMA": "IN": ["home", "house","housing"]] |
| current | "label": "HOUSING_INSTABILITY", "pattern": ["LEMMA": "not", "LEMMA": "able", "OP": "?", "LEMMA": "IN": ["pay", "afford"], "LOWER": "IN": ["my","his","her","their","the","our","this","that","one"], "LEMMA": "IN": ["housing", "house", "apartment", "home", "rent", "mortgage"]] |
| current | "label": "HOUSING_INSTABILITY", "pattern": ["LEMMA": "IN": ["concern","worry"], "OP": "?", "OP": "?", "OP": "?", "LEMMA": "IN": ["pay", "afford"], "LOWER": "IN": ["my","his","her","their","the","our","this","that","one"], "LEMMA": "IN": ["housing", "house", "apartment", "home", "rent", "mortgage"]] |
| current | "label": "HOUSING_INSTABILITY", "pattern": ["LEMMA": "IN": ["hard", "difficulty", "trouble"], "OP": "?", "OP": "?", "LEMMA": "IN": ["pay", "afford"], "LOWER": "IN": ["my","his","her","their","the","our","this","that","one"], "LEMMA": "IN": ["housing", "house", "apartment", "home", "rent", "mortgage"]] |
| current | "label": "HOUSING_INSTABILITY", "pattern": ["LEMMA": "IN": ["no", "not"], "OP": "?", "LEMMA": "have", "LEMMA": "plan", "OP": "?","LEMMA": "where", "LEMMA": "to","LEMMA": "IN": ["sleep", "stay", "live", "move","reside","wander"]] |
| current | "label": "HOUSING_INSTABILITY", "pattern": ["LOWER": "housing", "LEMMA": "IN": ["insecure","insecurity"]] |
| current | "label": "HOUSING_INSTABILITY", "pattern": ["LOWER": "IN": ["wo", "without"], "LEMMA": "permanent", "LOWER": "IN": ["home", "house", "housing"]] |
| current | "label":"HOMELESSNESS","pattern":["LEMMA":"be","OP":"?","OP":"?","LEMMA":"homeless"] |
| current | "label":"HOMELESSNESS","pattern":["LEMMA":"find","LEMMA":"IN":["house","housing"],"LOWER":"for","LOWER":"IN":["pt","pat","patient"]] |
| current | "label":"HOMELESSNESS","pattern":["LEMMA":"IN":["homeless","houseless","unhoused"],"POS": "ADP","OP":"?","OP":"?","LEMMA":"IN":["street","shelter","motel","inn","car","vehicle","jeep","hostel","van"]] |
| current | "label":"HOMELESSNESS","pattern":["LEMMA":"IN":["live","lives","sleep"],"LOWER":"IN":["in"],"LOWER":"the","LEMMA":"IN":["street"]] |
| current | "label":"HOMELESSNESS","pattern":["LEMMA":"IN":["move","discharge"],"LOWER":"IN":["to","into"],"OP":"?","OP":"?","LEMMA":"IN":["shelter","motel"]] |
| current | "label":"HOMELESSNESS","pattern":["LEMMA":"IN":["return","back"],"LOWER":"IN":["to"],"LEMMA":"IN":["street","shelter"]] |
| current | "label":"HOMELESSNESS","pattern":["LEMMA":"IN":["return","back"],"LOWER":"IN":["to"],"LOWER":"IN":["the","homeless"],"LEMMA":"IN":["street","shelter"]] |
| current | "label":"HOMELESSNESS","pattern":["LEMMA":"IN":["return","discharge"],"OP":"?","OP":"?","OP":"?","OP":"?","OP":"?","OP":"?","OP":"?","LOWER":"to","OP":"?","LEMMA":"IN":["friend","grandmother","grandfather","grandmom","grandpa","parent","parents","sister","brother"],"OP":"?","LEMMA": "IN": ["home","house"]] |
| current | "label":"HOMELESSNESS","pattern":["LEMMA":"IN":["return","wander","reside","live","lives","stay","sleep","back"],"LOWER":"IN":["on"],"OP":"?","LEMMA":"IN":["street"]] |
| current | "label":"HOMELESSNESS","pattern":["LEMMA":"IN":["return","wander","reside","live","referral","refer"],"LOWER":"IN":["in","to","on","at"],"OP":"?","OP":"?","LEMMA":"IN":["shelter","motel","inn","hostel"]] |
| current | "label":"HOMELESSNESS","pattern":["LEMMA":"IN":["return","wander","reside","live","stay","sleep"],"OP":"?","LOWER":"IN":["in","to","on","at"],"OP":"?","OP":"?","LOWER":"path","LOWER":"of","LOWER":"life","LEMMA":"IN":["street","shelter"]] |
| current | "label":"HOMELESSNESS","pattern":["LEMMA":"IN":["sign","complete","signed","completion","discharge"],"OP":"?","LEMMA":"IN":["shelter","homeless"],"OP":"?","LEMMA":"IN":["checklist","paperwork","packet"]] |
| current | "label":"HOMELESSNESS","pattern":["LEMMA":"IN":["wander","reside","live","sleep"],"LOWER":"IN":["in","on"],"OP":"?","LOWER":"parking","LEMMA":"lot"] |
| current | "label":"HOMELESSNESS","pattern":["LOWER":"lives","LOWER":"IN":["in","to","on","at"],"OP":"?","OP":"?","LEMMA":"IN":["shelter","motel"]] |
| current | "label":"HOMELESSNESS","pattern":["LOWER":"unemployed","OP":"?","LEMMA":"homeless"] |
| current | "label":"HOMELESSNESS","pattern":["LOWER":"IN":["pt","pat","patient","he","she","i"],"LOWER":"has","LOWER":"no","LEMMA":"IN":["home","house","housing"],"OP":"?","LEMMA":"IN":["live","stay","reside","sleep"]] |
| current | "label":"HOMELESSNESS","pattern":["LOWER":"IN":["pt","pat","patient","he","she","i"],"LOWER":"IN":["do","does"],"LOWER":"not","LOWER":"have","LEMMA":"IN":["home","house","housing"],"OP":"?","LEMMA":"IN":["live","stay","reside","sleep"]] |
| current | "label":"HOMELESSNESS","pattern":["LOWER":"IN":["pt","pat","patient","he","she","i"],"LOWER":"IN":["don","doesn"],"LOWER":"t","LOWER":"have","OP":"?","OP":"?","LOWER":"IN":["home","house","housing"],"OP":"?","LEMMA":"IN":["live","stay","reside","sleep"]] |
| current | "label":"HOMELESSNESS","pattern":["LOWER":"IN":["pt","pat","patient","he","she","i"],"TEXT":"IN":["doesnt","dont"],"LOWER":"have","OP":"?","OP":"?","LOWER":"IN":["home","house","housing"],"OP":"?","LEMMA":"IN":["live","stay","reside","sleep"]] |
| current | "label":"HOMELESSNESS","pattern":["TEXT":"REGEX":"(pa?t\|patient\|he\|she)","LEMMA":"be","OP":"?","OP":"?","OP":"?","OP":"?","OP":"?","LEMMA":"homeless"] |
| current | "label":"HOMELESSNESS","pattern":["TEXT":"REGEX":"(pa?t\|patient\|he\|she)","OP":"?","OP":"?","TEXT":"REGEX":"(has\|have)","TEXT":"REGEX":"(be\|been\|being)","LOWER": "to","OP":"?","OP":"?","LEMMA":"shelter"] |
| current | "label":"HOMELESSNESS","pattern":["TEXT":"REGEX":"(pa?t\|patient\|he\|she)","OP":"?","OP":"?","TEXT":"REGEX":"(in\|on\|within)","LEMMA":"shelter"] |
| current | "label":"HOMELESSNESS","pattern":["TEXT":"REGEX":"(pa?t\|patient\|he\|she)","OP":"?","OP":"?","TEXT":"REGEX":"(transport(s?\|ed\|ing)\|transportations?)","LOWER": "to","OP":"?","OP":"?","LEMMA":"shelter"] |
| previous | "label": "HOMELESSNESS", "pattern": ["LEMMA": "has", "OP": "?", "LOWER": "no","LOWER":"IN":[ "where","place"], "OP": "?", "OP": "?", "LEMMA": "IN": ["stay", "sleep", "live"]] |
| previous | "label": "HOMELESSNESS", "pattern": ["LEMMA": "has", "OP": "?", "LOWER":"IN":[ "nowhere","noplace"], "OP": "?", "OP": "?", "LEMMA": "IN": ["stay", "sleep", "live"]] |
| previous | "label": "HOMELESSNESS", "pattern": ["LEMMA": "move", "OP": "?", "OP": "?", "OP": "?", "LEMMA": "friend", "OP": "?","OP": "?" ,"LOWER": "house", "LOWER": "to", "LOWER": "another"] |
| previous | "label": "HOMELESSNESS", "pattern": ["LEMMA": "no","LEMMA":"IN": ["where","place"], "LEMMA": "to", "LEMMA": "IN": ["stay", "sleep", "live"]] |
| previous | "label": "HOMELESSNESS", "pattern": ["LEMMA": "IN": ["sleep","stay", "live", "move","reside","wander"],"LOWER":"IN":["in","to","on","at"],"OP": "?","OP": "?","OP": "?","LEMMA": "IN": ["shelter", "hostel", "motel", "car", "vehicle","boat","street"]] |
| previous | "label": "HOMELESSNESS", "pattern": ["LEMMA": "IN": ["sleep","stay", "live","reside"],"LOWER": "out","LOWER":"of","OP": "?","OP": "?","LEMMA": "IN": ["shelter","car", "vehicle", "boat"]] |
| previous | "label": "HOMELESSNESS", "pattern": ["LEMMA":"IN": ["nowhere","noplace"], "LEMMA": "to", "LEMMA": "IN": ["stay", "sleep", "live"]] |
| previous | "label": "HOMELESSNESS", "pattern": ["LOWER": "never", "LEMMA": "have","OP": "?","OP": "?","LEMMA": "IN": ["housing","house","home"]] |
| previous | "label": "HOUSING_INSTABILITY", "pattern": ["LEMMA": "can", "LEMMA": "not", "OP": "?", "LEMMA": "IN": ["pay", "afford"], "OP": "?", "OP": "?", "LEMMA": "IN": ["housing", "house", "apartment", "home", "rent", "mortgage"]] |
| previous | "label": "HOUSING_INSTABILITY", "pattern": ["LEMMA": "concern", "OP": "?", "OP": "?", "OP": "?", "LEMMA": "IN": ["pay", "afford"], "OP": "?", "OP": "?", "LEMMA": "IN": ["housing", "house", "apartment", "home", "rent", "mortgage"]] |
| previous | "label": "HOUSING_INSTABILITY", "pattern": ["LEMMA": "lose", "OP": "?", "LEMMA": "IN": ["home", "house"]] |
| previous | "label": "HOUSING_INSTABILITY", "pattern": ["LEMMA": "not", "LEMMA": "able", "OP": "?", "LEMMA": "IN": ["pay", "afford"], "OP": "?", "OP": "?", "LEMMA": "IN": ["housing", "house", "apartment", "home", "rent", "mortgage"]] |
| previous | "label": "HOUSING_INSTABILITY", "pattern": ["LEMMA": "IN": ["hard", "difficulty", "trouble"], "OP": "?", "OP": "?", "LEMMA": "IN": ["pay", "afford"], "OP": "?", "OP": "?", "LEMMA": "IN": ["housing", "house", "apartment", "home", "rent", "mortgage"]] |
| previous | "label": "HOUSING_INSTABILITY", "pattern": ["LEMMA": "IN": ["housing", "house", "apartment", "home", "place"], "LEMMA": "IN": ["inadequate", "unsatisfactory", "inappropriate"],"OP": "?"] |
| previous | "label": "HOUSING_INSTABILITY", "pattern": ["LEMMA": "IN": ["look","seek"],"LOWER": "for","LOWER": "low","LOWER": "income","LOWER": "housing"] |
| previous | "label": "HOUSING_INSTABILITY", "pattern": ["LEMMA": "IN": ["no", "not"], "OP": "?", "LEMMA": "have", "LEMMA": "plan", "OP": "?","LEMMA": "where", "LEMMA": "to","LEMMA": "IN": ["sleep", "stay", "live", "move","reside","wander"]] |
| previous | "label": "HOUSING_INSTABILITY", "pattern": ["LOWER": "housing", "LEMMA": "issue","LOWER": "yes"] |
| previous | "label":"HOMELESSNESS","pattern":["LEMMA":"be","OP":"?","LEMMA":"homeless"] |
| previous | "label":"HOMELESSNESS","pattern":["LEMMA":"find","LEMMA":"IN":["house","housing"],"LOWER":"for","LOWER":"IN":["pt","pat","patient","him","her"]] |
| previous | "label":"HOMELESSNESS","pattern":["LEMMA":"IN":["go","move","look","discharge"],"LOWER":"IN":["to","into"],"OP":"?","OP":"?","LEMMA":"IN":["shelter","motel"]] |
| previous | "label":"HOMELESSNESS","pattern":["LEMMA":"IN":["homeless","houseless","unhoused"],"POS": "ADP","OP":"?","OP":"?","LEMMA":"IN":["street","shelter","motel","inn","boat","car","vehicle","hostel"]] |
| previous | "label":"HOMELESSNESS","pattern":["LEMMA":"IN":["return","go","discharge"],"OP":"?","OP":"?","OP":"?","OP":"?","OP":"?","OP":"?","OP":"?","LOWER":"to","OP":"?","LEMMA":"IN":["friend","grandmother","grandfather","grandmom","grandpa","parent","parents","sister","brother"],"OP":"?","LEMMA": "IN": ["home","house"]] |
| previous | "label":"HOMELESSNESS","pattern":["LEMMA":"IN":["return","wander","reside","live","stay","sleep","back","referral","refer"],"LOWER":"IN":["in","to","on","at"],"OP":"?","OP":"?","LEMMA":"IN":["street","shelter","motel","inn","boat","hostel"]] |
| previous | "label":"HOMELESSNESS","pattern":["LEMMA":"IN":["return","wander","reside","live","stay","sleep","go","move","place"],"OP":"?","LOWER":"IN":["in","to","on","at"],"OP":"?","OP":"?","LOWER":"path","LOWER":"of","LOWER":"life","LEMMA":"IN":["street","shelter"]] |
| previous | "label":"HOMELESSNESS","pattern":["LEMMA":"IN":["sign","complete","signed"],"OP":"?","LEMMA":"IN":["shelter","homeless"],"OP":"?","LEMMA":"IN":["checklist","paperwork","packet"]] |
| previous | "label":"HOMELESSNESS","pattern":["LEMMA":"IN":["wander","reside","live","stay","sleep"],"LOWER":"IN":["in","on","at"],"OP":"?","OP":"?","LOWER":"parking","LEMMA":"lot"] |
| previous | "label":"HOMELESSNESS","pattern":["LOWER":"lives","LOWER":"IN":["in","to","on","at"],"OP":"?","OP":"?","LEMMA":"IN":["street","shelter","motel"]] |
| previous | "label":"HOMELESSNESS","pattern":["LOWER":"IN":["pt","pat","patient","he","she","i"],"LOWER":"has","LOWER":"no","LEMMA":"IN":["home","house","housing"]] |
| previous | "label":"HOMELESSNESS","pattern":["LOWER":"IN":["pt","pat","patient","he","she","i"],"LOWER":"IN":["do","does"],"LOWER":"not","LOWER":"have","LEMMA":"IN":["home","house","housing"]] |
| previous | "label":"HOMELESSNESS","pattern":["LOWER":"IN":["pt","pat","patient","he","she","i"],"LOWER":"IN":["don","doesn"],"LOWER":"t","LOWER":"have","OP":"?","OP":"?","LOWER":"IN":["home","house","housing"]] |
| previous | "label":"HOMELESSNESS","pattern":["LOWER":"IN":["pt","pat","patient","he","she","i"],"TEXT":"IN":["doesnt","dont"],"LOWER":"have","OP":"?","OP":"?","LOWER":"IN":["home","house","housing"]] |
| previous | "label":"HOUSING_INSTABILITY","pattern":["LEMMA":"go","LOWER":"to","OP":"?","LOWER":"assisted","LOWER":"living"] |

Table S3. Examples of homeless/housing insecurity descriptions in clinical notes.

| Category of description | Examples |
| --- | --- |
| Negated description | I’m not homeless;  patient is not considered homeless;  patient denied living on the streets;  patient denied having unstable housing |
| Nonpatient or general related description | patient volunteered at an event over the weekend to feed the homeless;  patient has a brother who is homeless;  patient now is working for homeless community;  homeless protocol and policy |
| History description | had been homeless in the past;  previously having lived in the streets for approximately 6 months;  was homeless several year ago;  homelessness 2016 |
| Positive/definite description | chief compliant pt is homeless;  patient is homeless living on the street;  is evicted and has no place to go;  can’t afford my mortgage |
